# Diffusion-Induced Ordered Nanowire Growth: Mask Patterning Insights

**DOI:** 10.3390/nano14211743

**Published:** 2024-10-30

**Authors:** Kamila R. Bikmeeva, Alexey D. Bolshakov

**Affiliations:** 1 Alferov University, Khlopina 8/3, St. Petersburg 194021, Russia; 2 Moscow Center for Advanced Studies, Kulakova Str. 20, Moscow 123592, Russia; 3 Laboratory of Advanced Functional Materials, Yerevan State University, Yerevan 0025, Armenia

**Keywords:** nanowires, epitaxy, selective area growth, modeling, kinetics

## Abstract

Innovative methods for substrate patterning provide intriguing possibilities for the development of devices based on ordered arrays of semiconductor nanowires. Control over the nanostructures’ morphology in situ can be obtained via extensive theoretical studies of their formation. In this paper, we carry out an investigation of the ordered nanowires’ formation kinetics depending on the growth mask geometry. Diffusion equations for the growth species on both substrate and nanowire sidewalls depending on the spacing arrangement of the nanostructures and deposition rate are considered. The value of the pitch corresponding to the maximum diffusion flux from the substrate is obtained. The latter is assumed to be the optimum in terms of the nanowire elongation rate. Further study of the adatom kinetics demonstrates that the temporal dependence of a nanowire’s length is strongly affected by the ratio of the adatom’s diffusion length on the substrate and sidewalls, providing insights into the proper choice of a growth wafer. The developed model allows for customization of the growth protocols and estimation of the important diffusion parameters of the growth species.

## 1. Introduction

Semiconductor nanowires (NWs) are promising structures for the development of nanophotonic, opto- and nanoelectronic devices, such as light-emitting diodes, semiconductor lasers, solar cells [1,2] and more intriguing applications such as NW thermoelectrics [3] and superconducting thermal switches [4]. Together with quantum dots [5], these quasi-one-dimensional nanostructures are known for their small footprint area which provides several advantages over planar structures including high crystalline perfection even on highly mismatched substrates [6,7]. Together with the vapor–liquid–solid (VLS) growth mechanism [8,9] which uses the growth catalyst, the NW geometry provides new pathways for the synthesis of unique nanoheterostructures with both axial and radial (core-shell) heterojunctions [10,11,12] and the even development of novel flexible semiconductor devices that cannot be obtained with 3D and planar structures [13].

Considering the nanoscale features of NW array-based nanoelectronic, optoelectronic or photovoltaic devices, it becomes evident that each individual NW functions as a distinct nanoscale device in its own right. So, for the development of scalable applications, such as efficient emitters with narrow bandwidths, typically, NWs with uniform physical properties and dimensions are needed [14]. Despite these nanostructures having several advantages for the fabrication and application of novel devices, few technological issues must be overcome. In its classical form, VLS uses catalyst film deposition over the unpatterned growth substrate, leading to the formation of arbitrarily distributed and shaped catalyst particles after the metal film annealing [15]. The latter, in turn, leads to the formation of disordered NW arrays with a sufficient dispersion of lateral sizes, making the synthesized NWs inapplicable in several cases. So, for the development of highly efficient devices based on NWs, it is important to obtain an ordered NWs array with a controlled diameter and spatial arrangement.

During the last decade, different approaches for selective area epitaxial growth (SAEG) were extensively developed. These included high resolution electron beam lithography (EBL) which suffers from high cost and poor scalability [16,17], and direct laser writing which provides acceptable resolution but still lacks production scalability [18]. Some novel methods including patterning for the selective epitaxy with the use of microsphere photolithography [19,20] and nanoimprint lithography [21] have been developed recently, leading to a rise in interest in SAEG in both academia and industry.

Recent works on the synthesis of semiconductor NWs with the aid of in-situ imaging shed light on growth peculiarities such as time scales in nucleation of a new layer and its growth [22]. The results strengthen the link between growth dynamics and the theoretical calculations aimed at growth control. The review in [23] examines critical technologies for synthesizing and assembling planar NWs, highlighting recent advances in their horizontal growth on crystalline or patterned substrates. Additionally, it discusses the advantages of achieving controlled geometries and compositions, along with applications for high-performance electronic devices such as field-effect transistors and photodetectors. Another recent review [24] discusses the remarkable potential of semiconductor NWs in various technological fields and categorizes their fabrication methods into top-down, bottom-up and hybrid approaches. It comprehensively evaluates the advantages of each synthesis paradigm while addressing critical aspects such as dimensional control, doping and the creation of unique NW structures tailored for specific applications.

The advancement of synthesis methodologies for the fabrication of ordered arrays of nanostructures necessitates a comprehensive theoretical investigation into the mechanisms underlying their formation. In most cases, specific theoretical investigations of the growth process consider the formation of a single NW without taking into account the influence of the other NWs on the kinetics of the species on the growth substrate [25]. But, with patterning technologies, one can control both the diameter of the growing nanostructures with the size of the mask openings and their position; in other words, the pitch between the neighboring NWs provides the ability to vary the overall morphology of the growing array. On the other hand, a change in the pattern morphology leads to a change in the growth species kinetics.

A study of the pitch influence on NWs SAEG was carried out in [26]. A model considering exponential decay of the adatom’s concentration with distance from NW on the substrate surface was introduced, and the influence of the pitch on the NWs elongation was analyzed both experimentally and theoretically. A similar effect of the pitch influence on the NW elongation was observed in MBE SAEG InAs NWs’ catalyst-free growth [27]. In [28] the authors studied the MBE growth of InAs NWs considering diffusion on the substrate and NW sidewalls but did not assume a change in the diffusion flux with time due to the redistribution of the growth species on the NW sidewalls. Previous studies also reported a diffusion-induced growth model of the ordered NWs’ array [29], lacking discontinuity. The growth of regular self-catalyzed GaP NW arrays was extensively studied in [30] to demonstrate that re-emission from the substrate surface plays a major role in this self-catalyzed process. This effect of the growth material secondary flux was modeled in [31].

While substantial progress has been made in the development of innovative approaches for the selective epitaxial growth of semiconductor NWs, significant gaps remain in our understanding of the underlying kinetics and interactions that govern their formation. The complexities arising from the influence of growth mask geometry, spacing arrangements and the dynamics of adatom diffusion necessitate a more comprehensive theoretical framework to guide the design and optimization of nanostructure arrays. This paper aims to bridge these gaps by presenting a detailed investigation of the formation kinetics of ordered NW arrays, emphasizing the critical role of pitch and growth mask configurations on diffusion dynamics. By elucidating these interactions and providing a predictive model for the growth protocols, we aspire to enhance the scalability and efficiency of NW-based devices, paving the way for future advancements in nanophotonics and nanoelectronics. Thus, our findings contribute not only to fundamental research but also to practical applications in the rapidly evolving field of semiconductor nanotechnology.

## 2. Model

**Figure 1:**
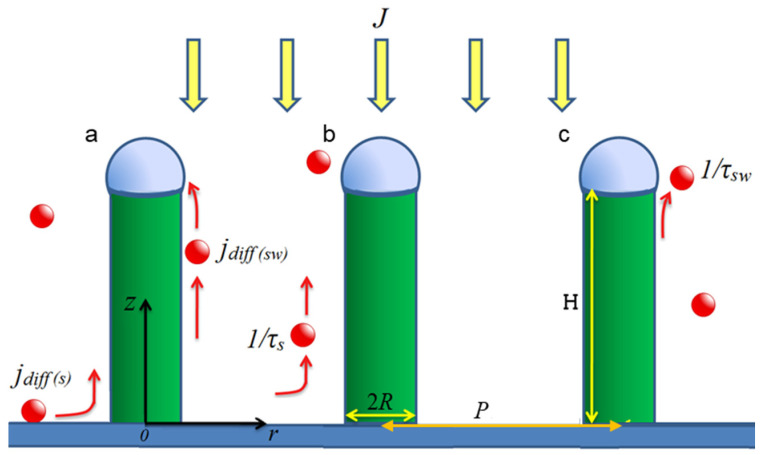
Schematic representation of the NW VLS growth. *J* is the growth flux promoting direct impingement of the growth species into a catalyst. Droplet (**a**) *j_diff(s)_* corresponds to the diffusion flux from substrate to a NW; *j_diff(sw)_*—diffusion flux on the NW sidewalls; (**b**) 1/*τ_s_* corresponds to the desorption rate with the adatom lifetime *τ_s_* on the substrate; *R*—NW radius; (**c**) 1/*τ_sw_* corresponds to the desorption rate with the adatom lifetime *τ_sw_* on sidewalls; *P*—pitch (NW-to-NW disatance); *H*—NW length.

NW growth generally involves several stages. First, the substrate is activated with a layer of catalyst material and then heated to a temperature above the eutectic melting point of the semiconductor and catalyst solution followed by the formation of the catalyst droplets. In this approach, we consider liquid droplets placed at the desired areas defined by the patterning technique. The growth material is then deposited at a growth temperature. We consider diffusion-induced growth with the species attaining the droplet in the following ways (refer to Figure 1): direct impingement from the vapor phase; diffusion of adatoms adsorbed on the sidewalls to the NW tip; diffusion of adatoms from the substrate surface along the sidewalls [32].

The main goal of our study is to theoretically obtain the effect of the substrate patterning geometry on the NWs growth. So, first, we consider an NW elongation *dH* as the product of the change in the number of particles *dN* that has reached the catalyst droplet per time unit *dt*:

(1) $$\frac{dN}{dt}=\frac{\pi R^{2}}{\Omega }\frac{dH}{dt},$$

where Ω is the volume per pair of atoms (for binary solutions) in the solid phase and *R* is the NW and catalyst droplet radius (for the sake of simplicity, we consider hemispherical droplet). The effect of the droplet shape can be easily taken into account with the geometrical coefficient governing the efficiency of the species adsorption from the vapor phase.

In the simplest case of the ballistic growth mode, species reach the droplet directly from the vapor or molecular beam with the corresponding rate V=JΩχ, where *J* is the molecular flux intensity and χ is the adsorption coefficient accounting for the adsorption probability. VLS growth of III-V NWs is known to be limited with the transport of nondiffusive group V species. Due to high group III elements’ diffusivity, their kinetics may govern the VLS growth [33]. Here, we consider only the growth flux and kinetics of the species limiting the growth, considering that the second growth element is in excess. In this case *dN* is proportional to the duration of the deposition process and droplet surface area, and the adsorption coefficient χ. Then, the NW growth rate is constant:

(2) $$\frac{dH}{dt}=V,$$

Thus, in the trivial case of the ballistic regime, the NW elongation rate is constant with time and does not depend either on the pitch of the mask pattern or on the diameter of the mask openings. A diffusion mode in which the adatoms are transported to the NW tip both from the substrate and the sidewalls is more realistic since diffusion fluxes may vary depending on the ratio between substrate and sidewall adatom diffusion length. In this case, the NW elongation equation must consider the diffusion.

## 3. Results

### 3.1. Diffusion on Substrate

In our model we used the following approximations:

- NWs are cylinders with constant radii (in time and along the NW length), while the NWs are positioned regularly on the substrate with the pitch *P* between the neighboring NWs (see Figure 1);
- The distribution of adatoms on the substrate around each NW has cylindrical symmetry (the closest case is hexagonal order of the NWs);
- The deposition flux is perpendicular to the substrate (incident angle can be easily considered with geometrical coefficients);
- The NW bottom perimeter and the catalyst droplet are ideal absorbers for the diffusing species.

With the listed assumptions, we are able to obtain the adatoms’ concentration distribution on the substrate around an individual NW and its sidewalls via the solutions to the diffusion equations, which are discussed below. To study the adatoms’ kinetics, we first analyze their distribution over the substrate in a steady state regime, neglecting the initial redistribution of the adatoms at the very beginning of the deposition process and assuming that the distribution is not affected by formation of the NWs. To calculate the distribution, we solve the diffusion equation:

(3) $$D_{s}\Delta n_{s}-\frac{n_{s}}{\tau_{s}}+J\chi_{s}=0,$$

where $D_{s}$—diffusion coefficient of the growth species on the substrate; $n_{s}$—corresponding adatom concentration; $\tau_{s}$—average adatom lifetime on the substrate before the desorption; J—impingement flux; and $\chi_{s}$—substrate adsorption coefficient, which is also attentive to the flux direction. This equation takes into account the species adsorption proportional to the flux intensity and desorption dependent on the average adatom lifetime. Expression (3) is the inhomogeneous Poisson equation. Following the approximation of the cylindrical symmetry of the concentration *n_s_* distribution, we conclude that this concentration is a sole function of the radius vector *r* (see Figure 1): *n_s_* = *n_s_*(*r*). Following the assumption that the NW footprint perimeter acts as an ideal absorber and considering that the maximum adatoms’ concentration is reached in-between the two NWs, we choose the following boundary conditions:

(4) $$\left\{\begin{array}{c} n_{s}{|}_{r=R}=0\\ n_{s}^{\prime }{|}_{r=\frac{P}{2}}=0 \end{array}\right.\;\;,$$

where *R* is the NW radius, and *P* is the pitch between two neighboring NWs. The solution to Equation (3) with boundary conditions (4) is the combination of modified Bessel functions of the first and second kind of zero orders:

(5) $$n_{s}\left(r\right)=n_{s}^{eq}\left({{1-C}_{1}I}_{0}\left(\frac{r}{\lambda_{s}}\right)-C_{2}K_{0}\left(\frac{r}{\lambda_{s}}\right)\right)\;\;,\;\left\{\begin{array}{c} C_{1}=K_{1}\left(\frac{P}{2\lambda_{s}}\right)f^{-1}\left(0,\frac{R}{\lambda_{s}},\frac{P}{2\lambda_{s}}\right)\\ C_{2}=I_{1}\left(\frac{P}{2\lambda_{s}}\right)f^{-1}\left(0,\frac{R}{\lambda_{s}},\frac{P}{2\lambda_{s}}\right) \end{array}\right.$$

where $\lambda_{s}=\sqrt{D_{s}\tau_{s}}$ is adatom diffusion length; $n_{s}^{eq}=J\chi_{s}\tau_{s}$ is equilibrium concentration; and $f\left(i,x,y\right)=I_{i}\left(x\right)K_{i+1}\left(y\right)+I_{i+1}\left(y\right)K_{i}\left(x\right)$.

**Figure 2:**
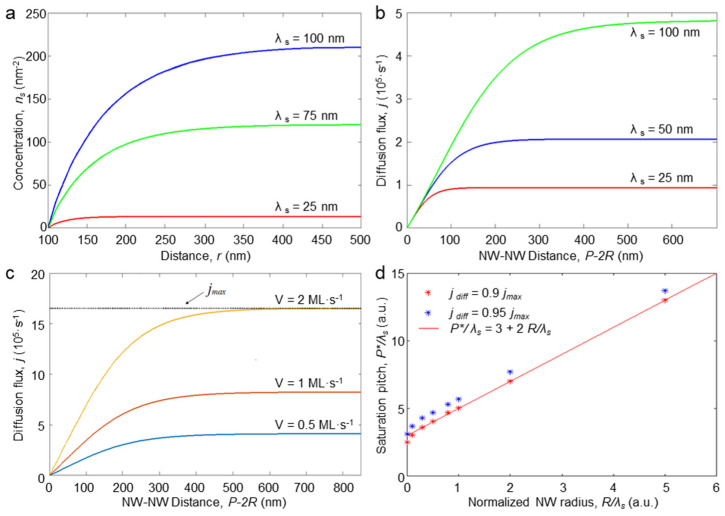
Modeling results: substrate diffusion. (**a**) Distribution of adatoms’ concentration on a substrate for different substrate diffusion lengths: *R* = 100 nm; *P* = 1000 nm; Ω = 0.056 nm^3^; *V* = 1 ML/s; $D_{s}$ = 250 nm^2^/s; *χ* = 1. Dependencies of the substrate diffusion flux on the distance between the NWs for different (**b**) substrate diffusion lengths calculated with the use of the parameters above and (**c**) impingement fluxes with the following parameters: *R* = 200 nm; $D_{s}$ = 250 nm^2^/s; Ω = 0.056 nm^3^; $\lambda_{s}$ = 100 nm. (**d**) Normalized over $\lambda_{s}$ saturation pitch dependence on normalized NW radius: red dot are values calculated for P* corresponding to 0.9 *j_max_*, blue are values for 0.95 *j_max_*, and the red line corresponds to the equation P* = 3$\lambda_{s}$ + 2*R*.

Figure 2a illustrates the radial distribution of adatoms around an NW towards the neighboring NW for different values of the adatom diffusion length and fixed diffusion coefficient. It can be seen that, as the NW acts as a sink for the adatoms, their concentration grows rapidly when moving from the NW at a distance comparable to the diffusion length. This means that the diffusion flux is limited with the migration of adatoms from the ring around the NW with a radius approximately the diffusion length. If the pitch is large enough, then the concentration saturates at $n_{s}^{eq}$. The latter value is the product of an equilibrium between the adsorption and desorption processes.

Next, to find the NW elongation rate, we analyzed the diffusion flux to the NW footprint, which is proportional to the gradient of the adatom concentration along the NW perimeter and is determined by the formula:

(6) $$j_{\;diff}=2\pi {RD}_{s}n_{s}^{\prime }{|}_{r=R}=\frac{2\pi {RD}_{s}n_{s}^{eq}}{\lambda_{s}}B\;,$$

where $B=C_{2}K_{1}\left(\frac{R}{\lambda_{s}}\right)-{C_{1}I}_{1}\left(\frac{R}{\lambda_{s}}\right)$. Figure 2b illustrates the dependence of the diffusion flux on the pitch for different values of the diffusion length on the substrate. The curves presented in Figure 2b confirm that the flux increases with the substrate diffusion length as well as with the impingement flux rate (Figure 2c). Clearly, in a situation with a close-packed geometry (*P* = *2R*), there is no diffusion flux as there is no free substrate surface. When the distance between the NWs reaches a certain value P*, the flux saturates at $j_{\;max}(R)=j_{\;diff}(R,P\to \infty \;)$. The P* value was estimated via finding a solution to the equation $j_{\;diff}(R,P*)=0.9j_{\;max}(R)$. Using Equation (6), the latter expression can be re-written in the following form:

(7) $$\frac{I_{1}\left(\frac{P*}{2\lambda_{s}}\right){\cdot K}_{1}\left(\frac{R}{\lambda_{s}}\right)-K_{1}\left(\frac{P*}{2\lambda_{s}}\right)\cdot I_{1}\left(\frac{R}{\lambda_{s}}\right)}{f\left(0,\frac{R}{\lambda_{s}},\frac{P*}{2\lambda_{s}}\right)}=0.9\frac{K_{1}\left(\frac{R}{\lambda_{s}}\right)}{K_{0}\left(\frac{R}{\lambda_{s}}\right)}\;\;,$$

The solution to this equation was found numerically and is plotted in Figure 2d. For the sake of comparison, the solution to $j_{\;diff}(R,P*)=0.95j_{\;max}(R)$ is also presented to demonstrate the proximity of the obtained P* values for the two different saturation values. In Figure 2d we also plot the P* = 3$\lambda_{s}$ + 2*R* linear dependence to demonstrate that it perfectly fits the obtained plot except for the very small values of *R* < $0.01\lambda_{s}$ where it rapidly decreases to 0. The obtained value accounts for a pitch corresponding to the saturation of the diffusion flux from the substrate towards the NW. It can be considered an optimum value in terms of the interplay between the NWs’ surface density and their elongation rate: denser arrays (smaller pitch) grow slower, while the elongation rate of the sparser arrays is saturated. This length depends on the NW’s radius and the adatoms’ kinetics ($\lambda_{s}$) and does not depend on the growth flux, which is clearly illustrated in Figure 2c.

### 3.2. Diffusion on Sidewalls

The diffusion of the growth species on the substrate governs the NW’s growth at its initial stage or if neither adsorption nor desorption occurs on the sidewalls. In other words, a ballistic diffusion regime governs the growth. The latter case seems illusive. That is why we proceeded to study the adatoms’ kinetics along the surface of the NW. The diffusion equation in that case is one-dimensional according to our model approximations (see Figure 1):

(8) $$D_{sw}\frac{d^{2}n_{sw}}{dz^{2}}-\frac{n_{sw}}{\tau_{sw}}+J\chi_{sw}=0,$$

where *D_sw_* is the diffusion coefficient on the sidewalls, *n_sw_* is the corresponding concentration of adatoms, *τ_sw_* is characteristic adatom lifetime on the sidewalls before desorption (we neglect the radial growth, therefore, the lifetime is determined only by the desorption), and *z* is axis directly perpendicular to the substrate surface with *z* = 0 corresponding to the substrate surface. To solve the equation, we adopt the following boundary conditions: (1) the droplet acts as a perfect species collector; (2) there is continuity in the diffusion flux along the NW footprint perimeter:

(9) $$\left\{\begin{array}{c} n_{sw}{|}_{z=H}=0\\ {D_{sw}n}_{sw}^{\prime }{|}_{z=0}=-{D_{s}n}_{s}^{\prime }{|}_{r=R} \end{array}\right.,$$

**Figure 3:**
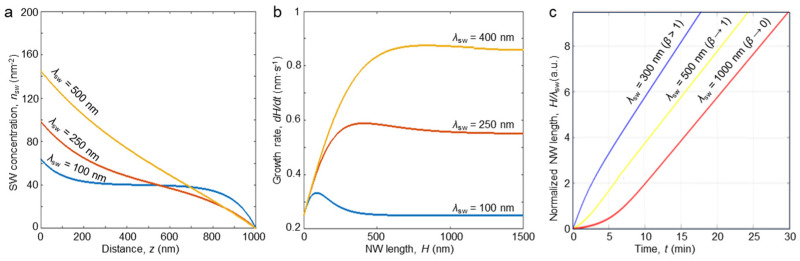
Modeling results: NW growth. (**a**) Distribution of adatom concentration on the sidewalls for different sidewall diffusion lengths (considering $R=100\;nm;\tau_{sw}=40\;s;Ω=0.05\;{nm}^{3},{V=0.05\;\frac{nm}{s};\;\lambda }_{s}=100\;nm;n_{s}^{\prime }{|}_{r=R}=0.24\;{nm}^{-3};\;and\;P=1000\;nm$). (**b**) Dependence of the growth rate on NW length *H* with the following parameters: $R=50\;nm;D_{sw}=\frac{200\;{nm}^{2}}{s};Ω=0.05\;{nm}^{3};V=0.05\;\frac{nm}{s},n_{s}^{\prime }{|}_{r=R}=0.5\;{nm}^{-3};\;and\;P=1000\;nm$. (**c**) Time dependence of the normalized NW length (*H*/$\lambda_{sw\;}$) for the different sidewall diffusion lengths and corresponding β values.

The solution to Equation (8) with boundary conditions (9) is the following:

(10) $$n_{sw}={\;\;n}_{sw}^{eq}\left(1-C_{3}\exp\left(\frac{z}{\lambda_{sw}}\right)-C_{4}\exp\left(-\frac{z}{\lambda_{sw}}\right)\;\right)\;\;\;,\;\left\{\begin{array}{c} C_{3}=\frac{A\lambda_{sw}exp\left(\frac{-H}{\lambda_{sw}}\right)+1}{2cosh(\frac{H}{\lambda_{sw}})}\\ C_{4}=\frac{-A\lambda_{sw}exp\left(\frac{H}{\lambda_{sw}}\right)+1}{2cosh(\frac{H}{\lambda_{sw}})} \end{array}\right.$$

where $\lambda_{sw}=\sqrt{D_{sw}\tau_{sw}}$ is diffusion length on the sidewall, and $n_{sw}^{eq}=J\chi_{sw}\tau_{sw},\;A=\frac{D_{s}{n_{s}^{\prime }|}_{r=R}}{D_{sw}n_{sw}^{eq}}$. Numerically calculated plots of the model adatom sidewall distribution for different diffusion lengths are presented in Figure 3a. The model predicts a monotonic decrease in the adatoms’ concentration towards the NW tip which is in agreement with the assumption of the catalyst droplet acting as a perfect sink. Importantly, Figure 3a unveils the intersection of the adatom concentrations characterized by different diffusion lengths. The explanation for this phenomenon is that, with a smaller diffusion length, the adatoms are less diffusive. As such, the concentration changes actively with smaller lambda values only in the bottom and top parts of a NW. Here the adatoms “feel” the influence of the substrate adatoms’ diffusion and a catalyst droplet acts as a perfect absorber. Meanwhile, in the middle part, the sidewall concentration is almost constant. The increase in the adatom diffusion length provides smooth diffusion along the sidewalls which is evident in Figure 3a for a 500 nm diffusion length. Here the enhanced diffusion is manifested by the monotonic decay of the adatom concentration towards the NW tip. This difference in the behavior of adatom kinetics provides the intersection of the concentrations at a distance of about 700 nm.

Next, we consider diffusion-induced elongation of a NW. In our model, the kinetic balance equation is:

(11) $$\frac{dN}{dt}=j_{diff(sw)}+J\chi \pi R^{2},$$

which corresponds to the elongation Equation (1). This allows us to define the change in the NW elongation rate depending on its length (Figure 3b) and, consequently, temporal dependence of NW’s length (Figure 3c). As can be seen, NW elongation can be sufficiently nonlinear during the initial growth stage due to the influence of the diffusion flux from the substrate which affects decays with time. At the beginning of the growth the elongation rate can be estimated as:

(12) $$\frac{dH}{dt}{|}_{H\to 0}=\frac{2D_{sw}\Omega {\;n}_{s}^{\prime }{|}_{r=R}}{R}+V,$$

Taking into account Expression (6), Equation (12) can be rewritten as

(13) $$\frac{dH}{dt}{|}_{H\to 0}=V\left(1+\frac{D_{sw}}{D_{s}}\frac{\chi_{s}}{\chi }B\;\frac{2\lambda_{s}}{R}\right),$$

When an NW grows larger, the substrate diffusion flux does not contribute to the vertical elongation and growth becomes time-independent. In this situation, growth occurs due to both the collection of adatoms from the sidewall surface near the NW tip at a distance of about *λ_sw_* due to the direct impingement:

(14) $$\frac{dH}{dt}{|}_{H\to \infty }=V\left(1+\frac{2\lambda_{sw}}{R}\right),$$

To quantitatively describe the nonlinearity of the NW formation in the initial growth stage, we introduce coefficient *β* (according to the previously reported results [34]), which predicts whether the elongation rate increases or decreases with time:

(15) $$\beta =\;\frac{D_{sw}}{D_{s}}\frac{\chi_{s}}{\chi }B\;\frac{\lambda_{s}}{\lambda_{sw}}\;\;,$$

In Figure 3c, we demonstrate nonlinearity of the NW formation depending on the value of β. When *β* = 0, due to a small $\lambda_{s}$/$\lambda_{sw\;}$ ratio, the diffusion flux from the substrate is small. Therefore, the growth at the initial growth stage is slow and is determined mostly by the direct impingement. In the opposite limiting case, when *β* > 1, an intense growth is first observed. However, with an increase in the NW length, the advantage of a large diffusion length on the substrate is leveled, and the dependence of vertical growth on time, as might be expected, becomes linear. An intermediate case of rather linear growth is *β*~1.

### 3.3. Experimental Data Fitting

**Figure 4:**
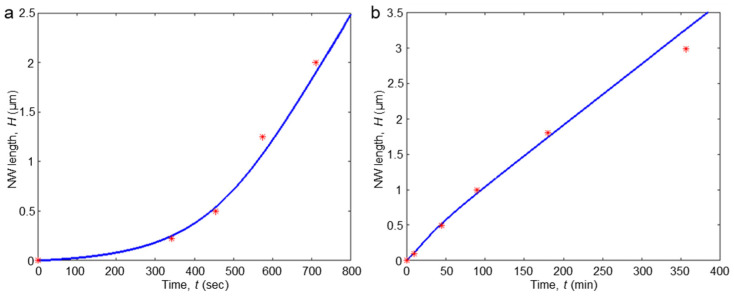
Experimental data fitting. (**a**) InP NW elongation experimental data obtained in [35]. Parameters of the experiment used in the modeling: deposition flux rate *V* = 0.04 nm/s, NW radius *R* = 12 nm, volume per atom pair Ω = 0.05 nm^3^. Fitting parameters: diffusion sidewall length $\lambda_{sw\;}$ = 1.0 μm, and diffusion coefficient $D_{sw}$ = 20 nm^2^/s. (**b**) InGaAs NW elongation data obtained in [27]; parameters of experiment: deposition flux rate *V* = 0.024 nm/s, radius *R* = 100 nm, pitch *P* = 1 μm, volume per atom pair Ω = 0.0556 nm^3^. Fitting parameters: diffusion sidewall length $\lambda_{sw\;}$ = 250 nm, diffusion coefficient $D_{sw}$ = 200 nm^2^/s.

In order to demonstrate the relevance and applicability of the developed diffusion-induced model, we numerically simulated the growth kinetics of the ordered NW arrays reported previously. In [35], the time dependence of InP NWs length was investigated via the formation of InAs markers at regular time intervals. The experimental data of NW length as a function of growth time and the fitting curve obtained via numerical simulation according to our model are plotted in Figure 4a. The fitting parameter values were the sidewall diffusion length $\lambda_{sw\;}$ = 1 μm and the diffusion coefficient $D_{sw}$ = 20 nm^2^/s. Similar kinetics were investigated in [27] with the InAs NWs array (see Figure 4b). The corresponding fitting parameters are diffusion sidewall length $\lambda_{sw\;}$ = 250 nm, and diffusion coefficient $D_{sw}$ = 200 nm^2^/s. Importantly, the obtained fitting parameters have reasonable values. The successful application of our diffusion-induced model to the growth kinetics of ordered nanowire arrays, as evidenced by the fitting of experimental data for both InP and InAs nanowires, underscores its robustness and relevance in realistic growth scenarios. The reasonable values of the fitting parameters—sidewall diffusion lengths and coefficients—align with expected physical behaviors and provide insights into the underlying mechanisms governing nanowire growth.

## 4. Conclusions

In this paper, we present a comprehensive model of the diffusion-induced formation of ordered NW arrays. Our analysis focuses on the migration of growth species on the growth substrate and the NW sidewalls, unveiling key dynamics that govern NW elongation. Our study reveals new insights into how the geometry of the growth mask significantly influences the dynamics of epitaxial NW formation. Understanding these geometric effects is crucial for tailoring the properties of NWs, thereby optimizing their performance in various semiconductor applications.

We have derived analytical expressions that facilitate the optimization of NW growth conditions. These expressions enable researchers to predict and control NW elongation rates based on specific growth parameters. This contribution serves as a valuable tool for both experimental and theoretical studies, improving the reproducibility and efficiency of NW fabrication processes. Our analysis of adatom diffusion on the substrate highlights the influence of neighboring NW pitch on growth dynamics, revealing that the diffusion flux initially increases with pitch before saturating at a critical value, P* = 3λ_s_ + 2*R*. This saturation point is key to optimizing NW elongation rates based on the material system in question.

We identify non-linearities in the NW growth rate during the initial stages. This insight prompts a re-evaluation of existing growth models and practices, emphasizing the need to consider such non-linear effects in future research to enhance the understanding and control of NW formation dynamics.

We demonstrate the applicability of our model to real-world NW growth scenarios, highlighting its potential to inform the development of tailored growth protocols. These protocols can enhance the uniformity and performance of semiconductor NWs, paving the way for advancements in device technology. The developed theoretical model aligns well with physical expectations and provides reasonable estimations for NW formation.

Overall, our findings significantly advance the understanding of NW formation mechanisms and offer practical insights for optimizing growth conditions in semiconductor NW technology. We believe that these contributions materially enhance the field and provide a robust framework for future research directions. The results correspond to kinetics of NW growth primarily governed by the diffusion processes of the growth species. This is a common characteristic shared by various growth methods making the results general.

Future work will aim to extend this model to explore more complex growth conditions and materials, including radial extension of the NWs and the effects of NW packing geometries (square and hexagonal arrangements), further solidifying its utility in advancing the field of nanostructure fabrication.

## Data Availability

Data are contained within the article.
